# Validation of an Ex Vivo Permeation Method for the Intestinal Permeability of Different BCS Drugs and Its Correlation with Caco-2 In Vitro Experiments

**DOI:** 10.3390/pharmaceutics11120638

**Published:** 2019-11-29

**Authors:** Aroha B. Sánchez, Ana C. Calpena, Mireia Mallandrich, Beatriz Clares

**Affiliations:** 1 Department of Pharmacy and Pharmaceutical Technology and Physical Chemistry, Faculty of Pharmacy and Food Sciences, University of Barcelona, 08028 Barcelona, Spain; aroha_89_1@hotmail.com (A.B.S.); anacalpena@ub.edu (A.C.C.); mireia.mallandrich@ub.edu (M.M.); 2 Department of Pharmacy and Pharmaceutical Technology, Faculty of Pharmacy, University of Granada, 18071 Granada, Spain

**Keywords:** Franz cells, Caco-2 cells, intestinal permeability, apparent permeability coefficient

## Abstract

The absorption study of drugs through different biological membranes constitutes an essential step in the development of new pharmaceutical dosage forms. Concerning orally administered forms, methods based on monolayer cell culture of Caco-2 (Caucasian colon adenocarcinoma) have been developed to emulate intestinal mucosa in permeability studies. Although it is widely accepted, it has disadvantages, such as high costs or high technical complexity, and limitations related to the simplified structure of the monolayer or the class of molecules that can be permeated according to the transport mechanisms. The aim of this work was to develop a new ex vivo methodology which allows the evaluation of the intestinal apparent permeability coefficient (P_app_) while using fewer resources and to assess the correlation with Caco-2. To this end, pig (*Sus scrofa*) duodenum segments were mounted in Franz diffusion cells and used to permeate four different drugs: ketorolac tromethamine (Kt), melatonin (Mel), hydrochlorothiazide (Htz), and furosemide (Fur). No statistically significant differences (*p* > 0.05) were observed corelating P_app_ values from Franz diffusion cells and Caco-2 cell experiments for Kt, Htz, and Fur. However, there were statistically significant differences (*p* < 0.05) correlating P_app_ values and Mel. The difference is explained by the role of Mel in the duodenal epithelial paracellular permeability reduction. Ex vivo permeation may be an equivalent method to Caco-2 for drugs that do not produce intestinal membrane phenomena that could affect absorption.

## 1. Introduction

**Figure 1:**
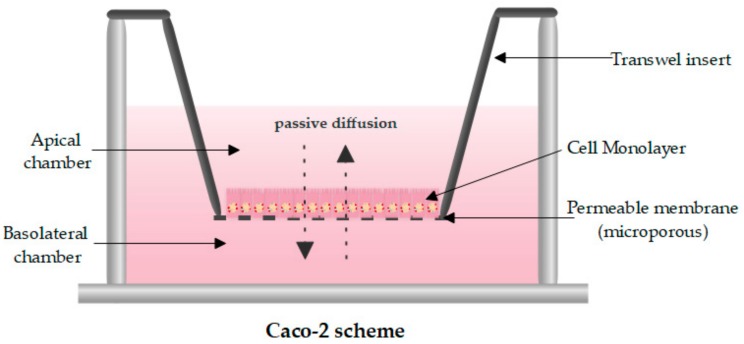
Caco-2 diagram formed by a transwell insert preloaded with polycarbonate membrane inserts with a known pore size. Original figure drawn in Edraw Max 9.4.

At the earliest stages of drug product or new pharmaceutical dosage form development, in vitro permeation through Caco-2 (human epithelial colorectal adenocarcinoma cell monolayer line) is widely accepted to estimate the intestinal apparent permeability coefficient (P_app_). As described in Figure 1, Caco-2 is a donor–receptor compartment apparatus separated by a cell monolayer grown on a porous polycarbonate filter. P_app_, defined as the flux of a substance permeating a membrane from the donor to receptor compartment normalized by the membrane surface and initial concentration in the donor chamber [1], is usually obtained based on a two-compartmental model approach; however, some authors have developed an alternative definition of a P_app_ index for three-compartment models describing the membrane as well as donor and receiver compartments [2]. This index may be predictive of oral bioavailability, showing an acceptable correlation with a human one, especially for drugs absorbed by passive diffusion. Carrier-mediated absorption drugs are not so easily extrapolated and require a scaling factor because of the low expression of carriers in this cell line [3]. Both undifferentiated and differentiated Caco-2 models have been developed, with the undifferentiated being more susceptible than the differentiated and, therefore, effective in cytotoxicity and cytoprotective studies [4,5,6,7,8]. More complex differentiated models are suitable for studying transport mechanisms and efficacy of substances [9,10,11,12].

Focusing on the relevance of Caco-2 in the design, optimization, and selection of potential candidates in the development of oral drugs, cell monolayer lines have been used for the study of improving the oral absorption of highly lipophilic and poorly water-soluble drugs [13]; lipid-based self-emulsifying drug delivery systems [14]; and for the evaluation of new oral formulations based on nanotechnology, such as solid lipid nanoparticles (SLNps) [15], or bioadhesive drug delivery systems, such as chitosan-modified nanoparticles [10]. Caco-2 has been optimized, although it exhibits variability attributable to biological methods [16], and the significance of emulating physiological conditions to improve in vitro experiments, for example, using bile acids, surfactants, or plasma proteins, is well known for providing a better in vitro–in vivo correlation [17]. Because of their usefulness and relevance, international institutions such the FDA accept in vitro permeability studies across Caco-2 to classify the permeability of drug substances according to the Biopharmaceutics Classification System (BCS) proposed in the International Council for Harmonization of Technical Requirements for Pharmaceuticals for Human Use (ICH) guidelines [18].

**Figure 2:**
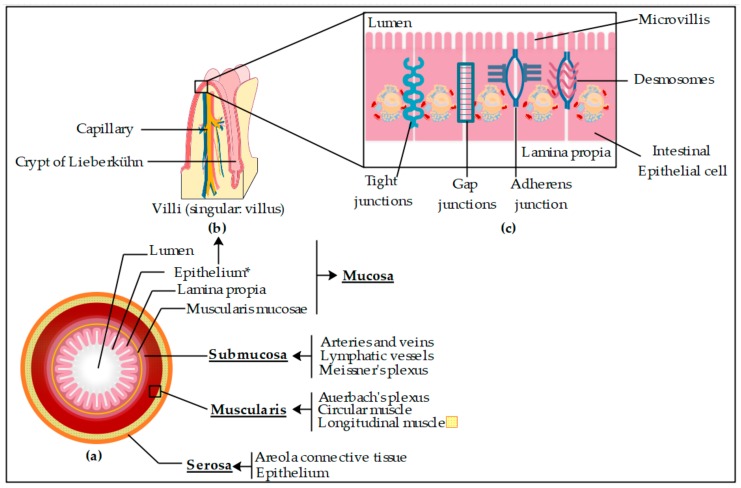
(**a**) Basic structure of small intestine layers, (**b**) a simplified schematic representation of villi, and (**c**) epithelium composed of intestinal epithelial cells including types of intercellular junctions. Original figure drawn from Edraw Max 9.4.

Regardless of it being an established method, the Caco-2 cell model has some disadvantages, such as its high cost or the need for highly specialized staff, which can be restrictive in both academic and private sector environments, where the optimization of resources is a paramount task. Other limitations are related to the structural and functional differences between a monolayer of cells and a biological membrane, such as the intestinal one. In contrast to Caco-2, which is a monolayer of cells, the gastrointestinal (GI) tract is composed of four main layers: tunica mucosa (mucous layer), tunica submucosa (submucous layer), tunica muscularis (muscle layer), and tunica serosa (serous layer) (Figure 2), which are additionally composed of different layers. Tunica mucosa is the most proximal layer to the lumen, which contains a thick layer of mucous covering the epithelial cells that are linked together by intercellular linkages, and an underlying layer called the lamina propria. The small intestine epithelium is a simple columnar epithelium, typical of regions of high secretion and absorption functions. The cells of simple columnar epithelium form finger-like projections called villi. In addition to intestinal villi, the surface of the cells contains microvilli, which collectively increase the surface area of the lumen by 400–600-fold [19].

Other important features of GI epithelium are the structural linkages between cells that connect the plasma membrane of neighboring cells. Four classes of intercellular junctions have been described: gap junctions (selective for small molecules such as ions, second messengers, and metabolites), tight junctions (paracellular barrier regulating the movement of water and solutes between epithelial layers), adherens junctions (help to seal the space between cells), and desmosomes (help to maintain shear forces and mechanical stress) (Figure 2c) [19].

GI membrane complexity demonstrates that drug absorption across this barrier is a multipathway process which could be classified as transcellular and paracellular. The most important is the transcellular route, whereby compounds go across the cells by traversing the cell membrane following passive diffusion or carrier-mediated transport (active transport, facilitated diffusion, absorption limited by P-glycoprotein or other efflux transports, intestinal first-pass metabolism followed by absorption of parent and metabolite- and receptor-mediated transport). There is also a paracellular passive diffusion via the junction route [20].

As explained above, the use of Caco-2, although predictive, is a very simplified approach to the GI membrane, so the use of ex vivo methods may lead to more accurate predictions of P_app_. Nejdfors et al. studied the permeability of C-mannitol, fluorescein isothiocyanate (FITC)–dextran 4400, a-lactalbumin, ovalbumin, and FITC–dextran through different intestinal regions of humans, rats, and pigs using small diffusion chambers of 5 mL and 1.74 cm^2^ of exposed tissue area (Navicyte, San Diego, CA, USA) [21]. Differences between intestinal regions and species were detected, and a good correlation between humans and pigs was also observed, mainly for the polyol mannitol in the jejunum and ileum. P_app_ was not compared with Caco-2 values, but the experiment showed that the use of intestinal membranes may be predictive of oral bioavailability.

Considering all the above, the aim of this work was to develop an alternative technique to Caco-2 and small diffusion cells, which allows the evaluation of the intestinal absorption rate or P_app_ for different drugs while using fewer resources and to assess the correlation with Caco-2. The purpose was to provide a new suitable and economical ex vivo method to test and compare new oral formulations or modified release systems, including therapeutic and higher drug concentrations than Caco-2.

## 2. Materials and Methods

### 2.1. Chemical and Reagents

Melatonin (Mel), ketorolac tromethamine (Kt), hydrochlorothiazide (Htz), ammonium dihydrogen phosphate (NH_4_H_2_PO_4_), phosphoric acid (H_3_PO_4_), disodium hydrogen phosphate (Na_2_HPO_4_), and tetrahydrofuran (THF) were purchased from Sigma-Aldrich (Madrid, Spain). Furosemide (Fur) was supplied by Acofarma (Barcelona, Spain). Potassium dihydrogen phosphate (KH_2_PO_4_), potassium hydroxide (KOH), acetonitrile (ACN), and methanol (MeOH) were purchased from Panreac Quimica (Barcelona, Spain). Hanks’ balanced salt solution (HBSS) was purchased from Merck S.L. (Barcelona, Spain). Double-distilled water was obtained from a Milli-Q^®^ Gradient A10 system apparatus (Millipore Iberica S.A.U., Madrid, Spain).

### 2.2. Instrumentation

#### HPLC-UV Procedure

The HPLC equipment consisted of a Waters^®^ Alliance 2695 Separation Module (Waters Co., Milford, MA, USA) with a 2996 Photodiode Array Detector (DAD) at a wavelength range of 190–800 nm and sensitivity settings from 0.0001 to 2.0000 absorbance units. HPLC parameters are summarized in Table 1 and listed below.

Kt analysis was conducted with a reverse-phase column C8 (150 × 2.1 mm) packed up with 5 µm (Kromasil^®^, Teknokroma Anlítica, SA; Barcelona, Spain), with a UV detector set up at 313 nm. The mobile phase, previously filtered by a 0.45 μm nylon membrane filter (Technokroma, Barcelona, Spain) and degassed by sonication, consisted of a 70:30 ratio of NH_4_H_2_PO (5.75 g/L; pH 3) to THF under isocratic elution at a flow rate of 0.25 mL/min. The injection volume was 10 μL.

Mel analysis was performed with a reverse-phase column C18 (150 × 4.6 mm) packed up with 3.5 µm (SunFire^®^, Waters Co., Milford, MA, USA), with a UV detector set up at 223 nm. The mobile phase, previously filtered by a 0.45 μm nylon membrane filter (Technokroma and degassed by sonication, consisted of a 55:45 ratio of double-distilled water to MeOH under isocratic elution at a flow rate of 0.6 mL/min. The injection volume was 100 μL.

Fur analysis was carried out with a reverse-phase column C18 (150 × 3.9 mm) packed up with 4 µm (Nova-Pack^®^, Waters Co., Milford, MA, USA), with a UV detector set up at 230 nm. The mobile phase, previously filtered by a 0.45 μm nylon membrane filter (Technokroma, Barcelona, Spain) and degassed by sonication, consisted of an 80:20 ratio of KH_2_PO_4_ (0.01M; pH of 6.3, adjusted with KOH 10%) to ACN under isocratic elution at a flow rate of 0.5 mL/min. The injection volume was 100 μL.

Htz analysis was conducted with a reverse-phase column ultrabase 100 ODS2 analytical column (100 × 4.6 nm; diameter of 3 µm (Akady, Spain)) with a UV detector set up at 224 nm. The mobile phase, previously filtered by a 0.45 μm nylon membrane filter (Technokroma, Barcelona, Spain) and degassed by sonication, consisted of a gradient elution of two solutions (A and B) at a flow rate of 0.7 mL/min. Solution A consisted of 940 mL of NaH_2_PO_4_ (35.8 g/L; pH of 3.2, adjusted with H_3_PO_4_) with 60 mL of MeOH and 10 mL of THF. Solution B consisted of 500 mL of NaH_2_PO_4_ (35.8 g/L; pH of 3.2, adjusted with H_3_PO_4_) with 500 mL of MeOH and 50 mL of THF. The percentage of B was 20% at time 0, 20% at 4 min, 80% at 10 min, 80% at 12 min, 20% at 13 min, and 20% at 20 min. The injection volume was 100 μL.

### 2.3. Validation and Verification of Analytical Methods

Previously validated HPLC-UV methods were selected for the analysis of the four assayed analytes. Considering that the samples were obtained from biological sources, the specificity was studied.

Specificity, expressed by the ICH guidelines as the ability to assess an analyte in the presence of components which may be expected to be present, was evaluated by the absence of interference of the phosphate-buffered saline (PBS; pH 7.4) and other components from biological membranes used as a blank at the retention times shown by the different standard solutions.

### 2.4. Ex Vivo Permeation Studies through Pig Intestine

#### 2.4.1. Franz Cell System and Intestinal Membrane

Ex vivo permeation was performed in the duodenum, the most proximal portion of the small intestine, of young female pigs (*Sus scrofa*). Animals were sacrificed for other purposes in the Animal Facility at Bellvitge Campus (University of Barcelona, Spain) (no. 7428) (Date of approval: 10 January 2019), and intestinal samples were obtained according to the 3R (reduction, refinement and replacement) principle. 

**Figure 3:**
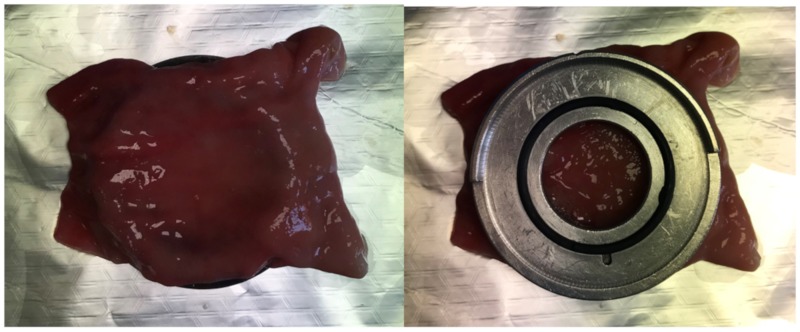
Piece of proximal small intestine of young female pigs (*Sus scrofa*) mounted on the metal ring of the Franz cells.

The duodenum was excised, cleaned, and preserved in HBBS at 5 ± 3 °C for 12 h. Then, 6 × 6 cm pieces were cut, mounted on the metal ring of the Franz cells as shown in Figure 3, and the remaining corners were trimmed.

To avoid damage to the biological intestinal membrane, 0.02 M PBS (pH 7.4) was prepared as a receiving medium. The composition was 0.6 g of KH_2_PO_4_ and 3.17 g of Na_2_HPO_4_ per liter of double-distilled water. The pH value was adjusted with H_3_PO_4_ or NaOH.

**Figure 4:**
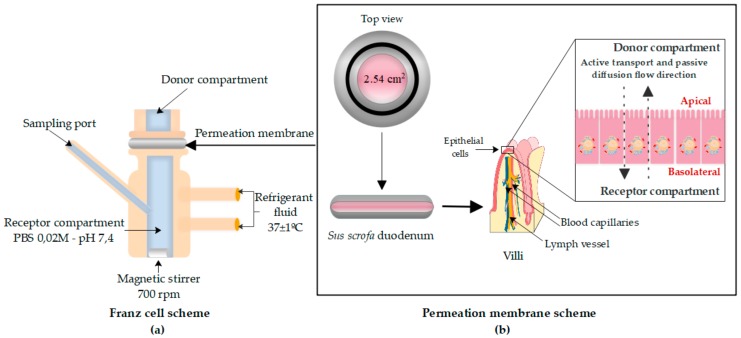
Franz cell scheme (**a**), including permeation membrane model (**b**) formed by the proximal small intestine of young female pigs (*S. scrofa*) that were opened with an incision and positioned with the area corresponding to the microvilli of the enterocytes in contact with the donor compartment and the basolateral part in contact with the receptor compartment. Original figure drawn in Edraw Max 9.4.

The ex vivo permeation study was performed in Franz diffusion cells (Vidra Foc Barcelona, Spain), where duodenum portions were placed between the receptor and donor compartments with the basal side in contact with the receiving medium and the apical side in contact with the donor chamber, avoiding bubble formation. The diffusion area was 2.54 cm^2^. A representative chart of the Franz cell system is shown in Figure 4.

Homogeneity during experiments was ensured by a small Teflon^®^-coated magnetic stir bar at 700 rpm. The diffusion cells were previously incubated in a water bath to equalize the temperature in all cells (37 ± 1 °C).

#### 2.4.2. Donor Solution Preparation and Sampling Method

Four drugs according to BCS classification were randomly selected. Table 2 includes the chemical structure; pKa (negative base 10 logarithm of the acid dissociation constant (Ka) of a solution) values; and tested formulations, including solvents, drug concentration, and pH.

All the drugs were dissolved by stirring at 30 ± 0.1 °C in PBS (pH 7.4) to guarantee the biocompatibility to the permeation membrane. Infinite dose conditions were ensured in all experiments. The donor compartment was then sealed by parafilm to prevent water evaporation. All the experiments were carried out under sink conditions, ensuring that the drug concentration in the receptor compartment was negligible compared to the donor one.

Samples of 300 µL were collected via a sampling port from the middle of the receptor compartment at preselected time intervals (30, 60, 90, 120, 180, 240, 300, 360, and 420 min) for 7 h. The removed sample volume was immediately replaced with the same volume of tempered fresh receiving medium of each molecule with great care to avoid trapping air beneath the membrane.

#### 2.4.3. Sample Analysis

The cumulative amount of the different BCS drugs through the small intestine membrane from the acceptor compartment was monitored by a validated HPLC-UV methodology. Results are reported as mean ± SD of five experiments for each drug.

#### 2.4.4. Data Treatment and Statistical Analysis

Our permeability model has the same structure as the two-compartment classic model, composed of donor (apical) and receptor (basal) chambers, both separated by the permeation membrane. So, apical-to-basal P_app_ was calculated based on classic parameters according to Equation (1):

P_app_ = (dQ/dt) / (C_0_ x A) (1)

where (dQ/dt) is the transport rate or flux (J) (µg/min) across the biological membrane, C_0_ (µg/mL) is the initial concentration of the drug in the donor chamber, and A is the surface area (cm^2^) of the permeation membrane.

The cumulative amount (Q) (µg) permeated through porcine duodenum was obtained by multiplying the acquired concentration (µg/mL) of each drug at the receptor chamber and the volume (mL) of the receptor chamber. J (µg/min) was calculated as the slope at the steady state obtained by linear regression analysis (GraphPad Prism^®^ software, v. 5.01, GraphPad Software Inc., San Diego, CA, USA) of Q as a function of time (min). Then, P_app_ (cm/min) was calculated according to Equation (1) by dividing the J (µg/min), the permeation area (A) (2.54 cm^2^), and the initial drug concentration (C_0_) (µg/mL = µg/cm^3^) in the donor chamber. Finally, the units were expressed in centimeters per second for comparison with the obtained results in the Caco-2 experiments. It was assumed that under sink conditions, the drug concentration in the receptor compartment is negligible compared to the donor compartment.

Obtained experimental data were analyzed by unpaired Student’s *t*-test to compare P_app_ values for both bibliographic Caco-2 results and experimental data obtained with Franz cells. A *p*-value < 0.05 was established as an indicator of statistically significant differences.

## 3. Results and Discussion

### 3.1. Obtained Kinetics Parameters and P_app_ Calculation

Table 3 shows the kinetics parameters of Kt, Mel, Fur, and Htz. Cumulative permeated drug was measured. Then, the flux and flux normalized by the permeation area (2.54 cm^2^) were calculated.

**Figure 5:**
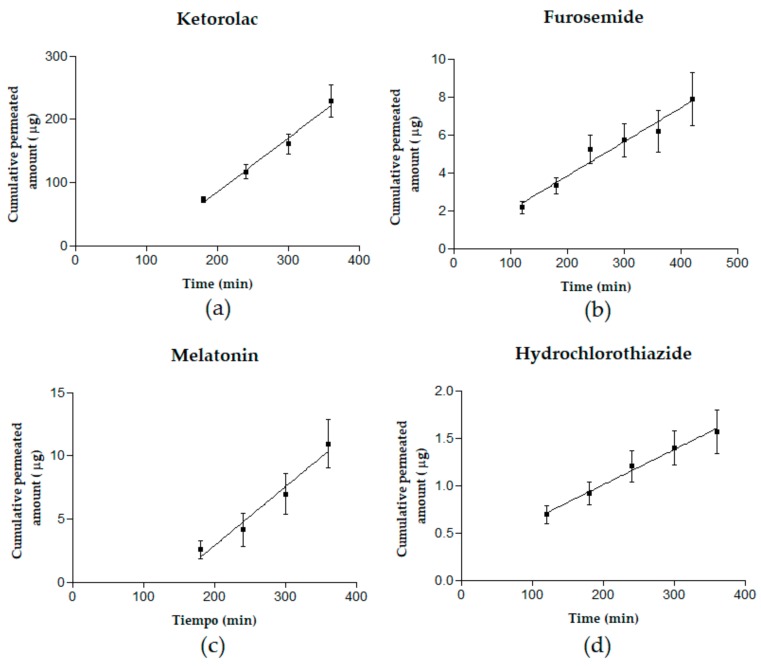
Cumulative permeated amounts (µg) as a function of time (min) of ketorolac tromethamine (**a**), melatonin (**b**), furosemide (**c**), and hydrochlorothiazide (**d**). Results are reported as mean ± SD (*n* = 5).

Figure 5 shows Kt, Mel, Htz, and Fur cumulative permeated amounts in micrograms as a function of time (min).

### 3.2. Specificity

Under the assay conditions described in the methodology section for each analyte, the mean retention times of Kt, Fur, Mel, and Htz were 9.45, 3.03, 5.05, and 9.5 min, respectively. The selectivity of the selected analytical method was confirmed by the studied chromatograms (Table S1), where Kt, Fur, Mel, and Htz peaks did not overlap with any other of the endogenous components of the medium. Blanks were obtained at time T_0_, from the receptor compartment, after incubation of diffusion cells and before adding the drugs. Therefore, the method is considered specific for the detection and quantification of the four molecules.

### 3.3. Correlation between P_app_ in Caco-2 versus Franz Cells

After a literature search, different Caco-2 permeation studies and P_app_ values were found for the tested drugs, which are summarized in Table 4.

**Figure 6:**
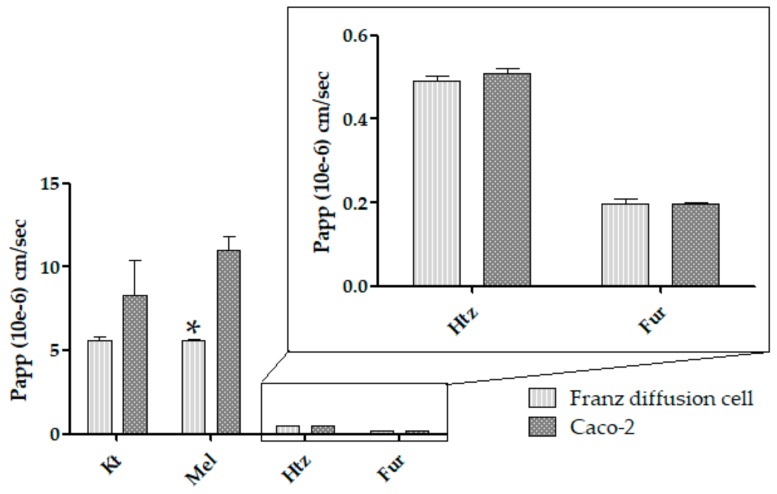
Comparative P_app_ between both Franz diffusion cells and Caco-2 cell culture for Kt, Mel, Htz, and Fur. Data are expressed as mean ± SD × 10^−6^ (cm/s). * *p* < 0.05.

Table 5 shows that no statistically significant differences (*p* > 0.05) were observed correlating Franz diffusion cells and Caco-2 cell experiments P_app_ values for Kt, Htz, and Fur. However, there were statistically significant differences (*p* < 0.05) correlating P_app_ values for Mel. Figure 6 shows a plot of statistical correlation.

P_app_ statistical correlation for both Franz diffusion cells and Caco-2 cell culture indicates that the ex vivo permeation is an equivalent method to Caco-2 for Kt, Htz, and Fur, which are BCS classes 1, 3, and 4, respectively. Regarding Mel, the obtained Franz diffusion cell P_app_ values showed statistically significant differences, with the ex vivo permeation data being 1.79 times lower than Caco-2. This could be related to the intestinal accumulation of Mel described in mammals [30], where Mel intestinal receptors MT1 and MT2 are involved in multiple roles, such as regulation of gastrointestinal motility and epithelial permeability [31]. It could also be explained by the fact that Mel, although it exhibits protein-facilitated transport [32], reduces the duodenal epithelial paracellular permeability [33]. This may justify the P_app_ value of 2.31 ± 0.12 × 10^−6^ cm/s (*n* = 4) for Mel obtained by other authors in an ex vivo permeation through rat jejunum in small diffusion chambers [34], which is also a lower and statistically different value than Caco-2. The difference may be also associated with the cytotoxicity in Caco-2 promoted by Mel concentrations of 1.56 and 0.78 µg/mL [35]. The ultrastructural damage in a simple structure such as a monolayer of cells would increase permeability through tight junctions, leading to an increased P_app_ value. Both circumstances would explain the differences. Drug solubility is not a limiting factor when applying Franz diffusion cells through an intestinal ex vivo membrane since Fur, which is BCS class 4, shows a good correlation. In contrast, intestinal membrane phenomena that modify intestinal permeation, such as accumulation or metabolism (among others), may hinder this method in the case of Mel.

## 4. Conclusions

A new ex vivo technique based on permeation through pig small intestinal membrane was developed. It allows the prediction of absorption rate or P_app_ and apical-to-basal permeation for different BCS drugs. This ex vivo method requires fewer economic resources than other in vitro techniques for P_app_ determination, providing a new suitable process to test and compare new oral formulations or modified release systems, including therapeutic and higher drug concentrations than Caco-2. Application of this method requires determining if the drug produces intestinal membrane phenomena that could affect the absorption process.

## Figures and Tables

**Table 1:** Summary of HPLC parameters for ketorolac tromethamine (Kt), melatonin (Mel), furosemide (Fur), and hydrochlorothiazide (Htz).

Molecule	Column	UV-ʎ (nm)	Mobil Phase	Flow Rate (mL/min)	IV^2^ (µL)
Kt	C8, 150 × 2.1 mm, 5 µm	313	NH_4_H_2_PO:THF (70:30)	0.25	100
Mel	C18, 100 × 4.6 mm, 3.5 µm	223	H_2_O:MeOH (55:45)	0.60	100
Fur	C18, 150 × 3.9 mm, 4 µm	230	KH_2_PO_4_:CAN (80:20)	0.50	100
Htz	100 ODS2, 100 × 4.6, 3 µm	224	NaH_2_PO_4_:MeOH:THF^1^	0.70	100

^1^ Hydrochlorothiazide HPLC mobile phase consisted of a gradient elution of two solutions: A (93:6:1) and B (47.6:47.6:4.8). ^2^ IV: injection volume.

**Table 2:** Name, structure, Biopharmaceutics Classification System (BCS) type, and pKa (negative base 10 logarithm of the acid dissociation constant (Ka) of a solution) of selected drugs for permeation experiments.

Molecule	Structure	BCS	pKa	Dissolution Media	Concentration
Ketorolac tromethamine	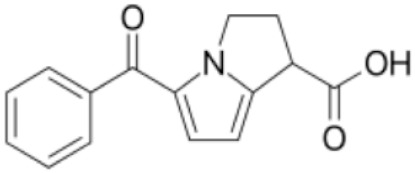	Class 1	3.5 [22]	PBS pH 7.4	1 mg/mL
Melatonin	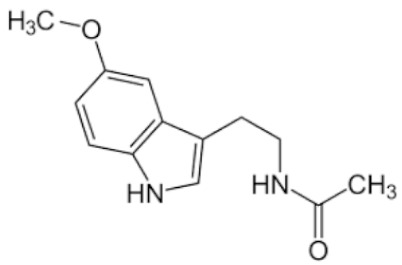	Class 2	16.5 [23]	PBS pH 7.4	0.8 mg/mL
Hydrochlorothiazide	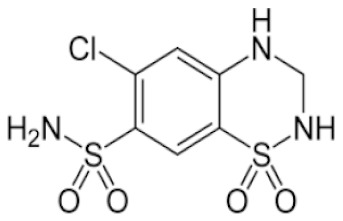	Class 3	7.9 [24]	PBS pH 7.4	0.05 mg/mL
Furosemide	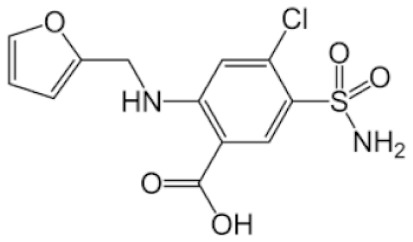	Class 4	3.9 [25]	PBS pH 7.4	0.6 mg/mL

**Table 3:** Permeation parameters for Kt, Mel, Fur, and Htz solutions in vertical Franz cells (*n* = 5).

Permeation Parameters	Kt	Mel	Htz	Fur
**Flux (µg/min)**	0.855 ± 0.069	0.683 ± 0.016	0.004 ± 0.0002	0.0180 ± 0.0018
**Flux/sup (µg/(cm/min))**	0.336 ± 0.027	0.268 ± 0.006	0.0015 ± 0.0001	0.0071 ± 0.0001
**Co (µg/mL)**	1000	800	50	600
**P_app_ (x10^−6^) (cm/s)**	5.609 ± 0.452	5.598 ± 0.130	0.487 ± 0.026	0.196 ± 0.020

Abbreviation: P_app_—apparent permeability coefficient.

**Table 4:** P_app_ values expressed as mean ± SD (cm/s) from different literature datasets about Kt, Mel, Fur, and Htz in Caco-2 experiments.

Drug	P_app_ (×10^−6^) (cm/s) (AP → BL)) Caco-2 Cells
Kt	8.30 ± 5.20 (*n* = 6) (HBBS^1^ pH 7.4) [26]
Mel	12.50 ± 0.01 (*n* = 3) (HEPES^2^ pH 7.4) [27]
Htz	0.51 ± 0.02 (*n* = 3) (HBBS^1^ pH 7.4) [28]
Fur	0.19 ± 0.01 (*n* = 3) (KBR^3^ pH 7.4) [29]

^1^ Hank’s balanced salts solution; ^2^ 4-(2-hydroxyethyl)-1-piperazineethanesulfonic acid^; 3^ Krebs–Ringer modified buffer (KBR).

**Table 5:** Statistical correlation between both Franz diffusion cells and Caco-2 experiments. P_app_ values are expressed as mean ± SD (cm/s) for Kt, Mel, Fur, and Htz.

Drug	P_app_ (×10^−6^) (cm/s) (AP → BL)		
	Franz Diffusion Cells (PBS pH 7.4)	Caco-2 (pH 7.4) ^1^	Unpaired *t*-Test (*p*)
Kt	5.61 ± 0.45 (*n* = 5)	8.3 ± 5.2 (*n* = 6) [26]	0.28 (*p* > 0.05)
Mel	5.60 ± 0.13 (*n* = 5)	12.50 ± 0.01 (*n* = 3) [27]	0.0001 (*p* < 0.05) *
Htz	0.49 ± 0.03 (*n* = 5)	0.42 ± 0.33 (*n* = 3) [28]	0.30 (*p* > 0.05)
Fur	0.20 ± 0.020 (*n* = 5)	0.19 ± 0.01 (*n* = 3) [29]	0.87 (*p* > 0.05)

^1^ Caco-2 experiments were carried out in Hank’s balanced salts solution (pH 7.4) for Kt and Htz, HEPES (pH 7.4) for Mel, and KBR pH 7.4 for Fur.

## Supplementary Materials

The following are available online at https://www.mdpi.com/1999-4923/11/12/638/s1, Table S1: HPLC-UV chromatograms of blank vs. standard solutions of ketorolac, furosemide, melatonin and Htz.
