# Supplementary material for: Validation of an Ex Vivo Permeation Method for the Intestinal Permeability of Different BCS Drugs and Its Correlation with Caco-2 In Vitro Experiments
Source: Pharmaceutics. 2019 Nov 29;11(12):638. doi: 10.3390/pharmaceutics11120638 (PMC6956211; doi:10.3390/pharmaceutics11120638)
Supplement: Supplementary file 1 [file pharmaceutics-11-00638-s001.pdf]

pharmaceutics

# Supplementary Materials: Validation of an Ex Vivo Permeation Method for the Intestinal Permeability of Different BCS Drugs and its Correlation with Caco-2 In Vitro Experiments

**Aroha B. Sánchez, Ana C. Calpena, Mireia Mallandrich and Beatriz Clares**

Selectivity of the selected analytical method was confirmed by the studied chromatograms, showed below, where ketorolac, furosemide, melatonin and hydrochlorothiazide peaks did not overlap with any other of the endogenous components of the medium. Blanks were obtained at time $T_0$, from the receptor compartment, after incubation of diffusion cells and before adding the drugs. Therefore, the method is considered specific for the detection and quantification of the four molecules.

**Table S1.** HPLC-UV chromatograms of blank vs. standard solutions of ketorolac, furosemide, melatonin and Htz.

| | | |
|---|---|---|
| **Ketorolac tromethamine** | **Blank** (Receptor medium PBS, pH 7.4) | 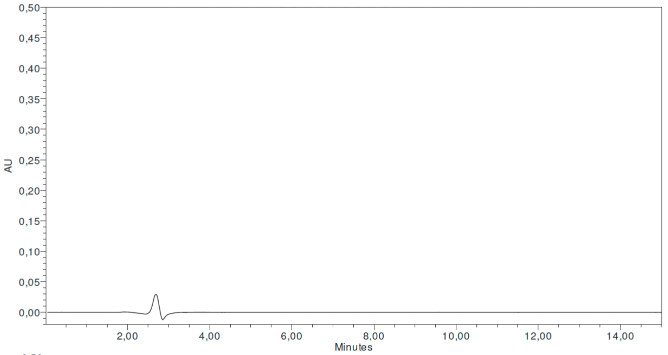  |
| | **Standard** (Rt Mean= 9.45) | 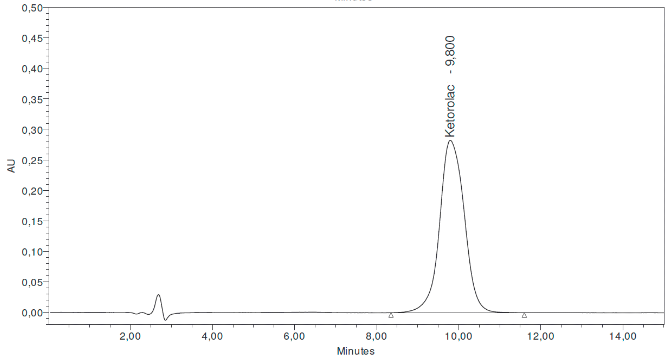  |

| | | |
|---|---|---|
| **Furosemide** | **Blank**<br><br>(Receptor medium PBS, pH 7.4) | 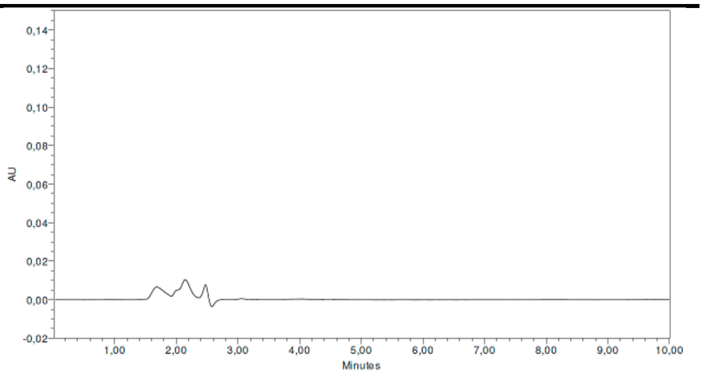 |
| | **Standard**<br><br>**(Rt Mean= 3.03)** | 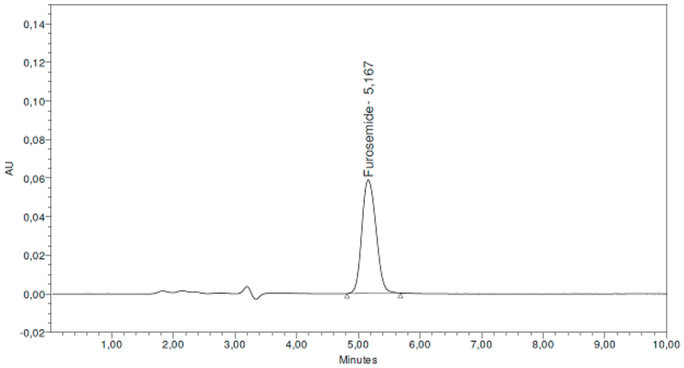 |

| | | |
|---|---|---|
| **Melatonin** | **Blank**<br><br>(Receptor medium PBS, pH 7.4) | 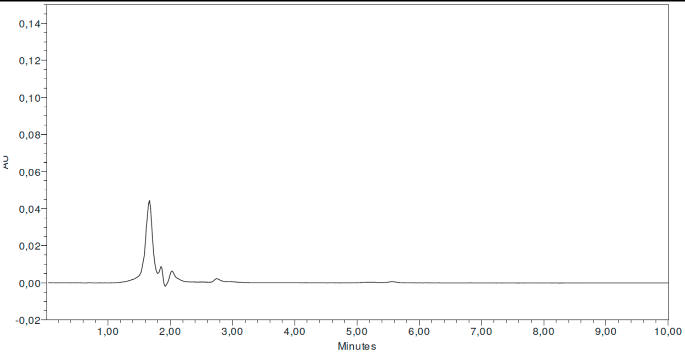 |
| | **Standard**<br><br>**(Rt Mean= 5.05)** | 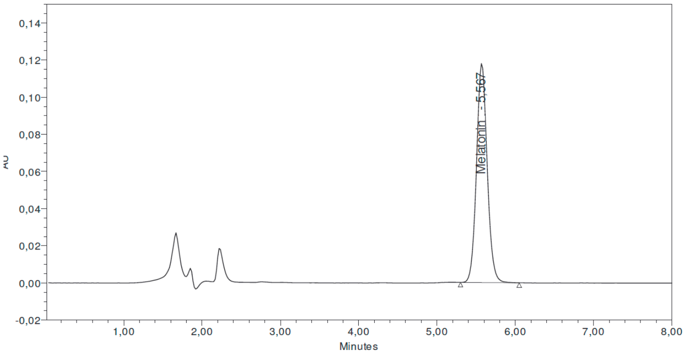 |

| | | |
|---|---|---|
| | **Blank**<br><br>(Receptor medium PBS, pH 7.4) | 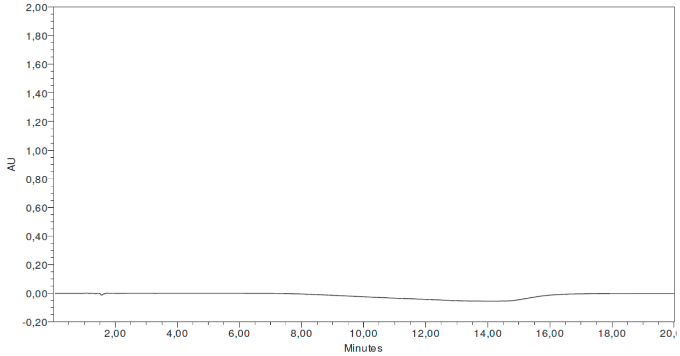 |
| **Hydrochlorothiazide** | **Standard**<br><br>**(Rt Mean = 9.5)** | 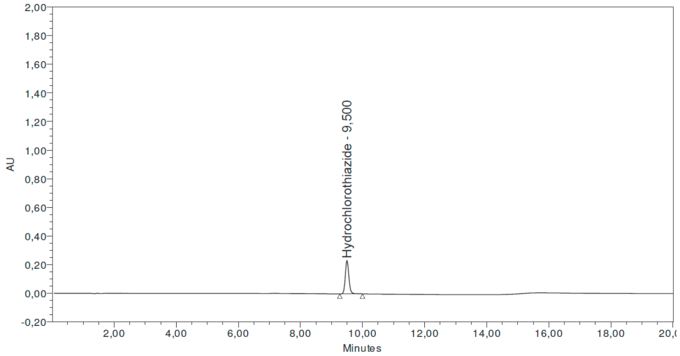 |
